# copick: An open dataset interface and toolkit for collaborative annotation and analysis of cryo‐electron tomography data

**DOI:** 10.1002/pro.70578

**Published:** 2026-04-17

**Authors:** Utz Heinrich Ermel, Jonathan Schwartz, Zhuowen Zhao, Daniel Ji, Ariana Peck, Yue Yu, Mohammadreza Paraan, Bridget Carragher, Achilleas S. Frangakis, Kyle I. S. Harrington

**Affiliations:** ^1^ Dynamic Structural Biology Biohub Redwood City California USA; ^2^ Buchmann Institute for Molecular Life Sciences and Institute for Biophysics Goethe University Frankfurt Frankfurt am Main Germany; ^3^ AI Research Biohub Redwood City California USA

**Keywords:** collaborative annotation, cryoET, cryo‐electron tomography, object detection, segmentation, structural biology, visual proteomics

## Abstract

Cryo‐electron tomography (cryoET) enables visualization of macromolecular complexes within intact cellular environments. Continued improvements in instrumentation, sample preparation, and data‐processing pipelines have increased both the scale and the complexity of cryoET datasets, making manual analysis challenging. To support scalable, collaborative annotation, we developed copick, an open‐source dataset application programming interface (API) and accompanying tool suite for cryoET analysis. Copick provides standardized access to tomograms, segmentations, point annotations, meshes, and feature maps across local storage, high‐performance computing systems, cloud platforms, and public repositories. Plugins for napari and ChimeraX enable human‐in‐the‐loop workflows for particle picking, segmentation, inspection of machine‐learning outputs, and project‐level collaboration. A multi‐resolution Open Microscopy Environment (OME)‐Zarr architecture supports responsive visualization and cross‐platform access. Copick additionally provides a Model Context Protocol interface enabling automated generation of annotation‐curation pipelines using natural‐language instructions. Together, these tools support reproducible, scalable, and collaborative cryoET analysis.

## 1 INTRODUCTION

Cryo‐electron tomography (cryoET) is a three‐dimensional (3D) imaging technique that allows reconstruction of cellular ultrastructure and macromolecular complexes in their native, near‐physiological state (Young & Villa, 2023). This method involves rapid freezing of biological specimens in vitreous ice, followed by collection of two‐dimensional (2D) images of the tilted specimen (a tilt series) using transmission electron microscopy and computational reconstruction of 3D tomograms. CryoET can provide sub‐nanometer resolution structural information while preserving the spatial relationships between cellular components, making it well suited for investigating protein complexes in their native cellular context (Kelley et al., 2024; Tegunov et al., 2021).

This method has been established as a valuable tool to study cellular architecture with molecular detail. Advances in sample preparation, as well as sample thinning using focused ion beam milling in scanning electron microscopes (FIB‐SEM) (Marko et al., 2007), have allowed researchers to apply cryoET to 100–300 nm thick sections of cells, tissues, and even whole organisms (Mahamid et al., 2015; Wang et al., 2012). Tilt series acquisition speed has been increased by improved automation and parallel collection schemes (Eisenstein et al., 2023, 2024; Klumpe et al., 2021). In combination with modern data acquisition and preprocessing pipelines, this now allows researchers to generate hundreds to thousands of 3D reconstructions of native cellular sections within days to weeks, resulting in dataset sizes that currently exceed the analysis capacity of a single researcher or even research group.

Some ubiquitous and high‐contrast biological structures, such as lipid bilayers and ribosomes, can already be reliably identified and labeled using automated computational methods, including template matching (Chaillet et al., 2023; Cruz‐León et al., 2024), image gradient‐based analysis (Martinez‐Sanchez et al., 2014), and pretrained neural networks (Lamm et al., 2024). However, segmentation of smaller complexes and membrane proteins remains a severe challenge. To achieve comprehensive visual proteomics, the ability to quickly identify and reconstruct a broad range of less abundant targets is required (Bäuerlein & Baumeister, 2021). This type of analysis still routinely requires manual or semi‐automated curation, and frequently performs best when using human‐in‐the‐loop active learning approaches and iterating on automatically generated annotations (de Teresa‐Trueba et al., 2023; Dobbs et al., 2025; Huang et al., 2024; Peck et al., 2025; Pyle et al., 2022).

Some aspects of these challenges are addressed in currently available software packages. Commercial tools like Amira (Stalling et al., 2005), DragonFly (Piché et al., 2017), and SyGlass (Pidhorskyi et al., 2018) integrate visualization, manual annotation, artificial intelligence (AI)‐driven segmentation, and segmentation proofreading. Among open‐source software packages, the ecosystem for this type of analysis remains more fragmented. Existing pipelines either do not include their own visualization and annotation capabilities, instead relying on popular tools like napari (Sofroniew et al., 2025) or ChimeraX (Meng et al., 2023), or use custom implementations with limited capabilities. Integration with data repositories and cloud infrastructure is limited in both the commercial and open‐source space. Additionally, combining workflows from different toolchains is hampered by the use of custom file formats and conventions, requiring time‐consuming development of conversion utilities. As a result, collaborative annotation and analysis of large cryoET datasets remain difficult.

These challenges motivated the development of copick, an open‐source dataset API for cryoET implemented in Python. Copick is accompanied by a set of tools, including plugins to the popular 3D image viewers napari (Sofroniew et al., 2025) and ChimeraX (Meng et al., 2023), as well as integration with large language model (LLM) based assistants. Here we describe the copick implementation and illustrate its application to the cryoET particle picking and segmentation tasks. Copick and related tools are available for download on GitHub (https://github.com/copick).

## 2 RESULTS

**FIGURE 1:**
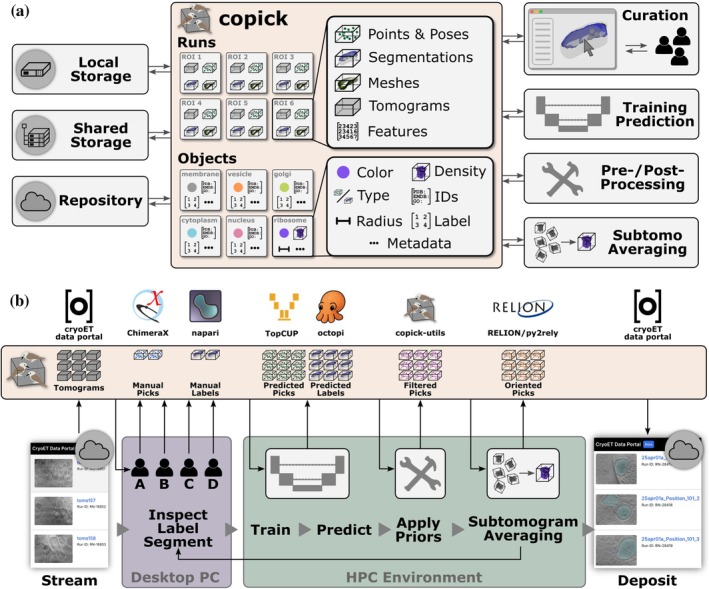
The architecture and size of copick datasets. (a) Data backing a copick project can be stored locally, on shared storage (e.g., an high‐performance computing (HPC) cluster or cloud storage server), or on the CZ cryo‐electron tomography (cryoET) data portal (Ermel et al., 2024). The set of data related to a single region of interest (ROI) is termed a *Run* and can include tomogram volumes, (oriented) point sets, voxel segmentations, triangular meshes, and *n*‐dimensional feature volumes. Objects to be annotated in each *Run* are defined along with default sets of parameters, including color, ontology terms, database identifiers, and in the case of protein complexes, expected radii and associated density maps. The copick toolkit exposes data for curation via ChimeraX and napari, allows pre‐ or postprocessing via a command‐line interface (CLI) and direct access via a python API for model training, prediction, or export to subtomogram averaging pipelines. (b) Shared copick projects allow building human‐in‐the‐loop workflows across compute and storage environments. Input tomograms can be streamed from the cryoET data portal and annotated on personal computers by multiple users simultaneously. Manual annotations of subsets of datasets serve as training data for copick‐enabled neural networks, such as octopi (Schwartz et al., 2026) and TopCUP (Zhao et al., 2026). Segmentations can be used as spatial priors using the copick toolkit, and ultimately select particles for processing in subtomogram averaging pipelines. Deposition of results to repositories is facilitated by integrated export commands.

Copick is an open‐source toolkit that enables multiple researchers to collaboratively pick particles, curate segmentations, and inspect machine‐learning predictions or template matching results on the same cryoET dataset, regardless of where the data are stored or which visualization software is used. At its core, a storage‐agnostic Python API provides unified access to tomograms, segmentations, point annotations, meshes, and feature maps across local filesystems, high‐performance computing (HPC) clusters, cloud platforms, and public repositories such as the CZ cryoET data portal (Ermel et al., 2024) (Figure 1b). Plugins for ChimeraX (Meng et al., 2023) and napari (Sofroniew et al., 2025) expose this functionality through familiar graphical interfaces, managing data organization and format conversions automatically so that researchers interact with annotations rather than files. A command‐line toolkit and accompanying Model Context Protocol (MCP) interface further allow annotation‐curation pipelines to be composed from natural‐language descriptions of biological constraints. Together, these components maintain data provenance across distributed teams while reducing the technical overhead typically associated with multi‐tool cryoET workflows.

### 2.1 The copick data model

Copick datasets are designed to allow quick iteration in machine learning development, training, and inference workflows through standardized data organization and simplified access to the data. Datasets support storage of and access to the five most commonly encountered data types in a typical cryoET annotation workflow: tomograms (3D volumes), volumetric segmentations, polygon meshes, point annotations, and *n*‐dimensional feature volumes (Figure 1a). Copick‐based datasets can span multiple storage locations, such as local and shared filesystems (e.g., using SAMBA, secure shell (SSH), or the file transfer protocol (FTP)), cloud storage like Amazon S3 or Google Cloud Storage, and public data repositories like the cryoET data portal (Ermel et al., 2024). The API abstracts the filesystem and data repository interfaces, allowing projects to include data from multiple sources and enabling unified access within a consistent data model. In addition, copick allows defining parameter sets for objects expected to be annotated in a project, including color, protein data bank (PDB) (wwPDB consortium, 2019) or electron microscopy database (EMDB) (The wwPDB Consortium, 2024) identifiers, gene ontology (The Gene Ontology Consortium, 2023a) terms, segmentation label, associated density maps, and other metadata. These parameter sets are respected throughout the toolkit.

Data stored in copick on disk are organized hierarchically, with tomograms and annotations derived from a single region of interest (i.e., reconstructed from the same tilt series) grouped into an entity termed a *Run* (as in an experimental run). Tomograms, segmentations and *n*‐dimensional feature vector volumes are stored based on their voxel sizes, while points and meshes are stored in angstrom coordinates. Poses are represented as rotation matrices. The matrix representation avoids ambiguities inherent in Euler angle conventions and provides a consistent intermediate format when converting between cryoET pipelines, where conversion through matrix representation is typically required regardless of the source convention.

A key advantage of copick is the ability to partition projects into static and mutable parts, called project layers. This feature allows multiple users to have common access to shared, read‐only portions of a dataset, while storing individual annotation results locally or in the cloud. Overlaid projects enable multiple researchers to easily collaborate on annotating or analyzing the same tomograms across organizations and infrastructure, without the risk of overwriting each other's work. The copick API provides methods to access and modify image and annotation data in a consistent manner, regardless of the underlying storage solution, reducing required boilerplate code for data access.

### 2.2 Multi‐scale image representation in copick

Tomograms reconstructed from cryoET tilt series typically range from 0.2 to 12 gigabyte (GB) per volume, depending on resolution and reconstruction dimensions. These file sizes present challenges for interactive visualization, as loading entire tomograms at full resolution causes delays that impede annotation throughput, especially when researchers must inspect many volumes. Network transfer of full‐resolution data from remote storage or HPC clusters further impacts latency. Additionally, storing thousands of tomograms at multiple resolution levels imposes substantial storage costs.

To address these challenges, copick stores all tomographic data in OME‐Zarr format (Moore et al., 2023), utilizing a chunked, compressed storage, and a three‐level resolution pyramid (see Section 3 for details). Downsampling levels are generated automatically during data import, though both chunk dimensions and pyramid parameters can be configured by users if needed. This approach reduces file sizes by ~10%. For example, a 1260 × 1260 × 492‐voxel float‐32 tomogram in MRC‐format (Cheng et al., 2015) is 3,124,397,824 bytes and in OME‐Zarr format is 2,860,707,522 bytes. With this reduction, the total size of pyramid‐resolution OME‐Zarr is only 5%–10% higher than that of a single‐resolution MRC‐file based on comparisons across three depositions on the CZ cryoET data portal (Ermel et al., 2024) (Figure S1A). The largest gain in storage efficiency is achieved in case of integer‐valued segmentations, which show compression ratios of 30:1 to 100:1 when applied to membrane segmentations available for the same three depositions at the highest resolution level (Figure S1B).

The multi‐scale representation enables optimizing data access patterns based on the curation tasks at hand. Low‐resolution data (4× binned) are usually sufficient for initial volume inspection and to confirm spatial relationships between particle picks and larger cellular structures such as organelles and membranes, which remain identifiable even at reduced resolution. High‐resolution data are accessed selectively for manual picking of challenging particles and neural network training. By maintaining all scales within a single file structure, tools can seamlessly transition between overview and detailed views without requiring separate file management or conversion steps.

### 2.3 Machine learning development infrastructure: copick‐utils and copick‐torch

Two companion libraries extend the core copick API with specialized functionality for annotation processing and deep learning workflows.

*copick‐utils* provides operations for annotation manipulation, including bidirectional format conversion between picks, segmentations, and meshes; Boolean and spatial filtering operations for enforcing geometric constraints; and processing functions such as connected component analysis, skeletonization, and convex hull generation. These operations are accessible through both a Python API and command‐line interface (see Table S1 for a full list of commands).

*copick‐torch* implements PyTorch‐compatible dataset classes that abstract the complexity of extracting training patches from multi‐resolution OME‐Zarr volumes, handling coordinate transformations between voxel and angstrom spaces, and managing class‐balanced sampling from imbalanced particle distributions. The library provides cryoET‐specific augmentations including Mixup for virtual training example generation and FourierAugment3D for frequency‐domain perturbations inspired by MemBrain (Lamm et al., 2024). Integration with MemBrain‐seg (Lamm et al., 2024) demonstrates how specialized models can be wrapped to operate on copick datasets.

Together, these libraries reduce typical particle detection and segmentation workflows, which normally require custom data loaders, augmentation pipelines, format converters, and constraint enforcement, to configuration of dataset parameters and composition of preprocessing operations. The standardized data organization ensures models trained on one dataset transfer directly to others without custom loading code, and integration with the cryoET data portal enables training datasets combining local expert annotations with large‐scale public data.

### 2.4 Human‐in‐the‐loop particle picking workflow using ChimeraX‐copick: collaborative particle picking, inspection, and active model training

While automated computational methods can reliably identify common structures such as membranes, the identification of less abundant complexes and membrane proteins remains challenging. Human‐in‐the‐loop active learning approaches, where researchers iteratively refine automatically generated annotations, have proven effective for these difficult cases (de Teresa‐Trueba et al., 2023; Huang et al., 2024; Pyle et al., 2022). The copick visualization plugins support this workflow by enabling inspection of machine learning outputs, manual correction of predicted annotations, refinement of particle orientations following subtomogram averaging, and incorporation of prior knowledge, such as high‐confidence segmentations.

To enable active learning workflows, we developed two ChimeraX plugins. The ChimeraX‐OME‐Zarr plugin (Ermel, 2025a) was developed to read tomograms and segmentations in OME‐Zarr format (Moore et al., 2023), which serves as the foundation for data visualization in ChimeraX‐copick. ChimeraX‐copick (Ermel, 2025b), built on top of the ChimeraX‐ArtiaX plugin (Ermel et al., 2022), provides an interface for collaborative particle annotation that supports visualization of tomograms, particle picks, volumetric segmentations, and mesh annotations.

Data management is handled automatically by the copick API, eliminating manual saving operations while allowing access to picks stored on remote filesystems, HPC clusters, cloud storage, or the cryoET data portal (Ermel et al., 2024). The copick data structure associates each point set, mesh, or segmentation with a pre‐configured object type that specifies color, particle radius, identity, and optional reference density maps. This configuration is defined once in the project setup rather than requiring per‐point‐set specification as in standalone ArtiaX (Ermel et al., 2022) workflows.

**FIGURE 2:**
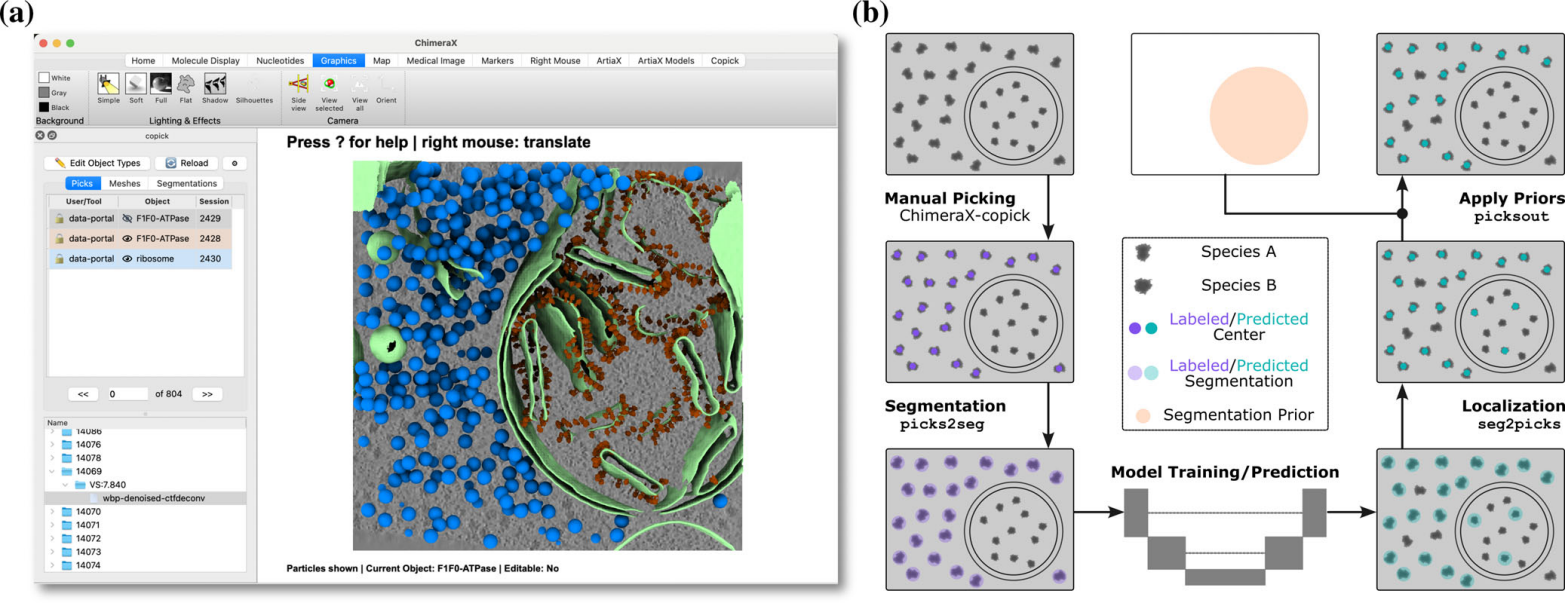
The ChimeraX‐copick interface and example cryo‐electron tomography (cryoET) object detection workflow. (a) A rendered scene of run 14,069 from dataset 10301 (Kelley et al., 2024) on the cryoET data portal in the ChimeraX‐copick interface. The plugin allows quick selection of tomograms and rendered objects in the left sidebar. Particles can be rendered as simple spheres (blue, ribosomes) or as an oriented surface (orange, F1Fo‐ATPase), along with voxel segmentations (light green, membranes). (b) Schematic of an example workflow encountered in cryoET object detection, and the copick tools implementing each step.

The ChimeraX‐copick interface displays available tomograms in an overview gallery and shows associated pick sets in a searchable sidebar panel, with entries color‐coded by object type and annotated with user and session identifiers to indicate provenance. Picks from static project layers (e.g., shared reference annotations or data portal picks) are displayed but protected from modification, though users can copy them to mutable layers for refinement (Figure 2a).

A typical picking workflow consists of launching the interface with a copick configuration file, selecting a tomogram from the gallery view or searchable run panel, opening an existing pick set or creating a new set for a specific object type, and placing picks. Switching between object types requires only selecting the corresponding pick set entry. In contrast, equivalent workflows in standalone ArtiaX (Ermel et al., 2022) require opening the tomograms and their corresponding coordinate files for each species manually and storing the new coordinates by multiple menu selections and mouse clicks. Beyond manual annotation, the interface facilitates inspection and validation of particle predictions from neural network models and of particle orientations following subtomogram averaging, supporting iterative human‐in‐the‐loop workflows.

The integration with the cryoET data portal (Ermel et al., 2024) further enables machine learning development at the community scale, leveraging a repository that now includes more than 20,000 publicly accessible tomograms along with more than 100,000 sets of annotations. Copick allows researchers to assemble training datasets that combine manually annotated tomograms from their own experiments with large‐scale predictions available through the portal. This approach enables models to benefit from high‐quality expert annotations and the diverse experimental conditions represented in portal data. In addition, copick's standardized data organization simplifies the implementation of data loaders for popular machine learning frameworks, reducing the development time required to train new models or adapt existing architectures to new particle types.

### 2.5 Collaborative volumetric segmentation in napari‐copick

napari‐copick (Harrington & Ermel, 2025) provides organized access to copick datasets that leverage napari's interactive painting support for annotation, enabling collaborative annotation workflows without reimplementing existing segmentation functionality (Sofroniew et al., 2025). The plugin interfaces directly with napari's native layer system, presenting tomograms as image layers, particle picks as points layers, and segmentations as labels layers, while the copick API manages file organization, multi‐resolution storage, and cross‐platform data access.

**FIGURE 3:**
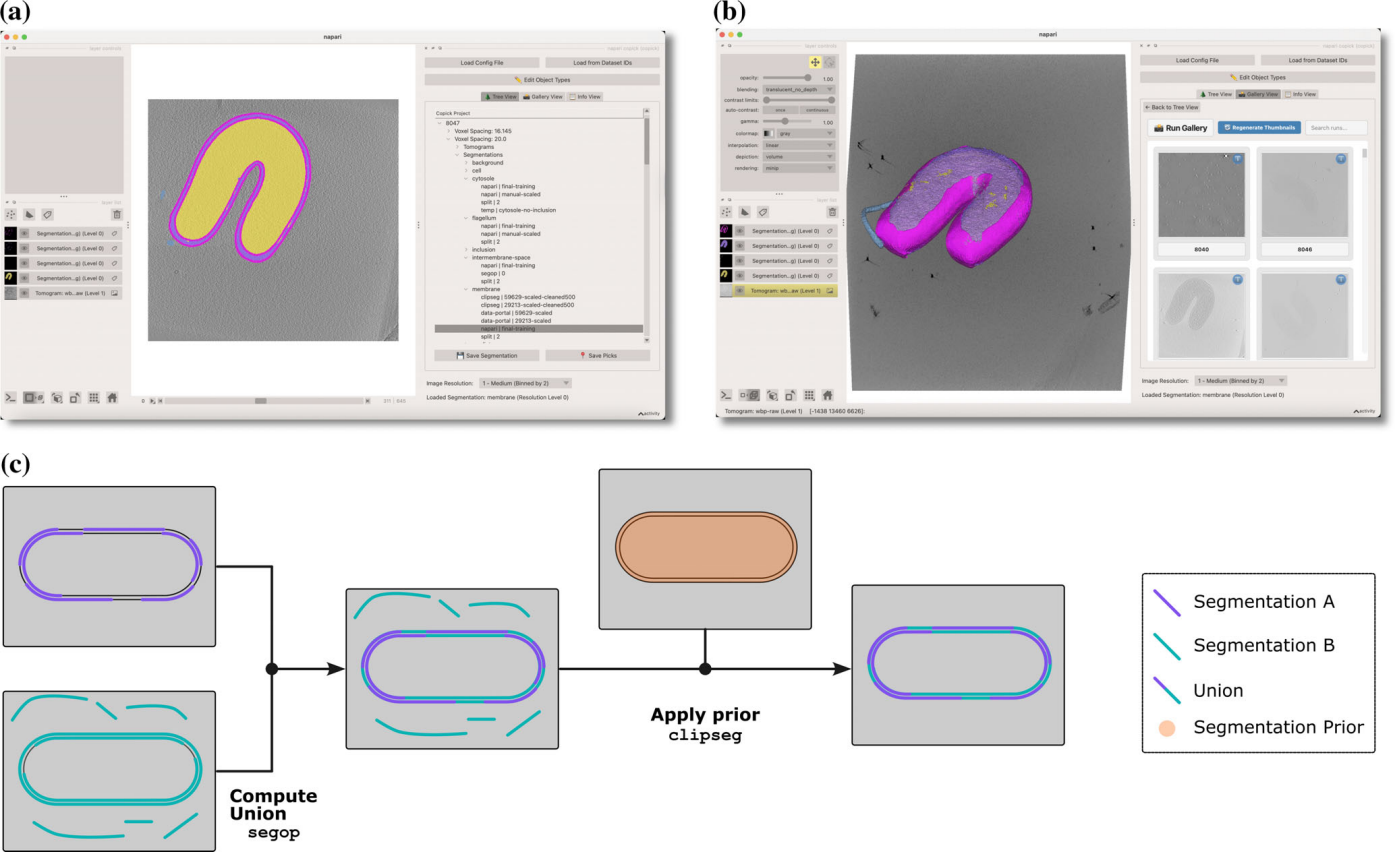
The napari‐copick interface and segmentation workflow. (a) An example tomogram from CZ cryo‐electron tomography (cryoET) data portal dataset 10155 (Kaplan et al., 2023b) is rendered as a 2D plane and a tree‐view of the copick project is shown in the right panel. Segmentations are colored according to values defined in the copick configuration file. (b) The tomogram is rendered as a three‐dimensional volume with minimum intensity projection; isosurfaces of the segmentations are displayed and the copick gallery view is shown in the right panel. (c) An example segmentation curation workflow and the copick tools implementing each step. Two automatically generated segmentations are combined to improve segmentation quality prior to applying prior information in the form of a distance limit to another segmentation's surface.

The interface re‐uses gallery view components from ChimeraX‐copick for tomogram selection (Figure 3b) and provides a hierarchical sidebar organizing runs, tomograms, and segmentations according to the copick data structure. Existing segmentations are displayed with user and session identifiers to indicate provenance, with color‐coding determined by object type definitions in the copick configuration (Figure 3a).

Segmentation workflows utilize napari's native painting, erasing, and 3D editing tools, but can also leverage recent machine learning‐aided methods like nnInteractive (Isensee et al., 2025). When saving, the plugin automatically scales the segmentation to the specified voxel spacing and writes multi‐resolution OME‐Zarr representations (Moore et al., 2023), eliminating manual resampling and format conversion steps.

### 2.6 MCP‐aided pipeline generation for annotation curation incorporating biological priors

The copick command‐line toolkit provides programmatic access to annotation processing operations organized into four functional categories: data processing (component separation, skeletonization, and size filtering), format conversion (bidirectional transformation between picks, segmentations, and meshes), logical operations (Boolean operations, spatial filtering, and containment testing), and project management. These operations can be composed to implement biological constraints expressible through spatial relationships and geometric properties (Figures 2b and 3c).

**FIGURE 4:**
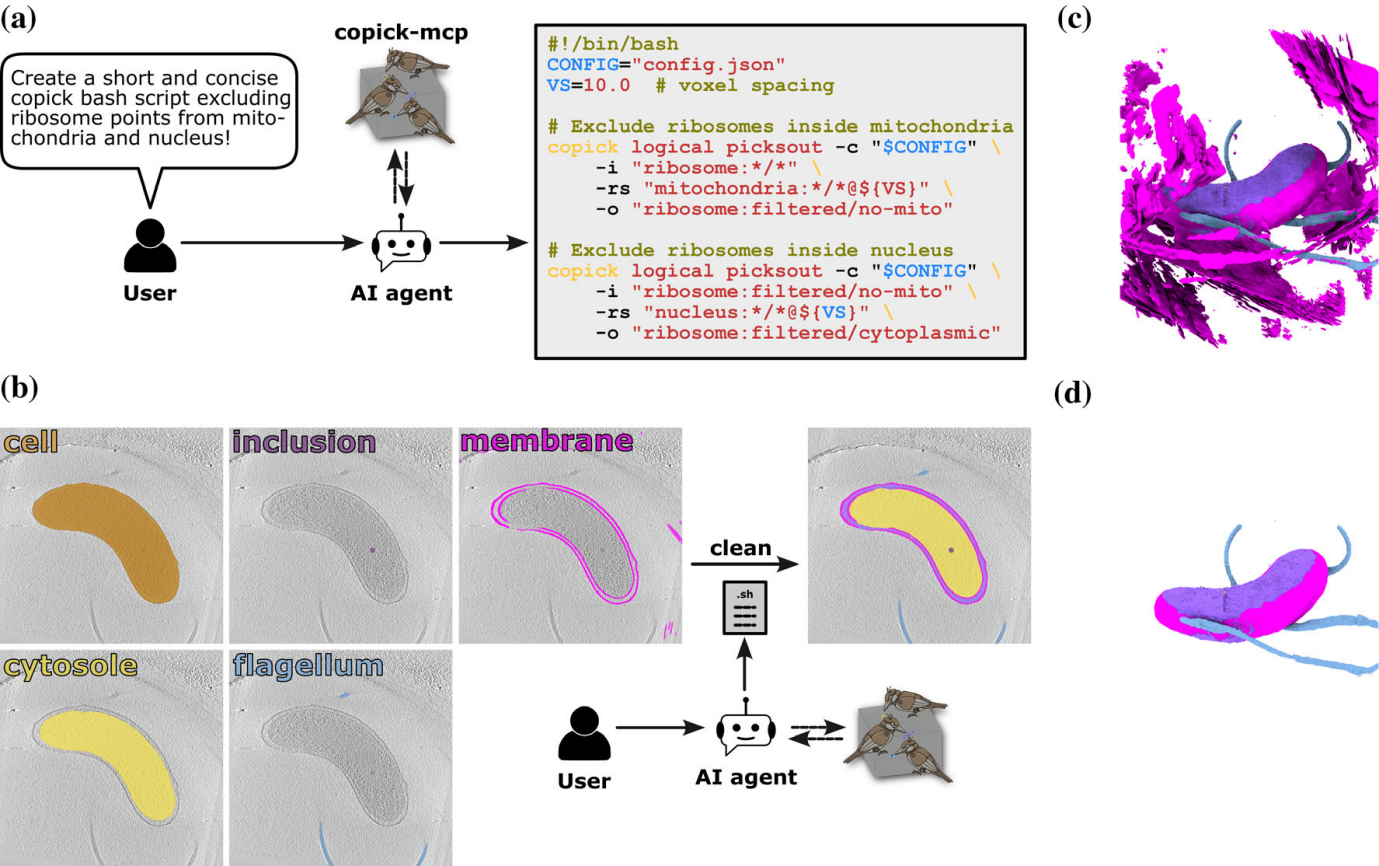
Pipeline generation aided by copick‐mcp. (a) Schematic of the typical workflow using copick‐mcp. Users provide natural language instructions to an AI agent with access to copick‐mcp. The agent queries copick's command‐line interface documentation at runtime and generates a syntactically correct bash‐script using the copick‐utils prototypes. (b) 2D slices of input and output segmentations of a complex segmentation curation and reconciliation task different from the example in (a) (see Table S1 for the full natural language prompt). Using geometrical constraints provided in natural language and copick‐mcp, the resulting pipeline was able to remove strong artefactual membrane labels from the input segmentation (c), yielding a curated membrane segmentation and previously non‐segmented intermembrane space (dark purple) (d).

To make these capabilities accessible without programming expertise, copick includes a MCP server that exposes command documentation and parameter specifications to LLMs through automated introspection. This enables translation of natural language descriptions of annotation workflows into executable bash pipelines (Figure 4a). Any biological prior expressible through spatial relationships, geometric properties, or logical combinations can be implemented by composing copick operations—for example, enforcing that particle picks lie within a specified distance of membrane surfaces (Figure 2b) or combining segmentations from multiple sources while applying distance constraints (Figure 3c).

As a demonstration, enforcing organelle topology constraints in bacterial cell segmentations required combining membrane segmentations from multiple sources, limiting annotations to regions near the cell surface, generating intermembrane space through Boolean subtraction, and ensuring non‐overlapping compartments (Figure 4b,c; detailed pipeline specification in Table S2). This approach eliminates the need to develop project‐specific validation tools for each biological system, as researchers can specify validation logic in natural language rather than code.

## 3 METHODS

### 3.1 Software implementation

The copick toolkit was implemented as a modular collection of open‐source Python packages released under the MIT license. The core API requires Python 3.10 or later and was tested on Linux, macOS, and Windows platforms. Filesystem interfaces are abstracted using the fsspec family of libraries, with backends for local filesystems, SSH (sshfs), and Amazon S3 (s3fs) included in regular testing. Data models and project configurations are defined using Pydantic schemas, enabling validation and type‐safe access to project parameters. Image data are stored according to the OME‐Zarr v0.4 specification (Moore et al., 2023). The command‐line interface was built using Click with a plugin architecture that allows external packages to register additional commands. Geometric operations on meshes and segmentations utilize trimesh, scikit‐image, and NumPy. Integration with the Chan Zuckerberg cryoET data portal is provided through the cryoet‐data‐portal client library (Ermel et al., 2024). Image import and export from or to MRC format is implemented using the mrcfile package (Burnley et al., 2017). Import and export of other cryoET file formats is facilitated by modules from the teamtomo family of packages, including starfile (Burt et al., 2025), dynamotable, and emfile. Implementation of the copick toolkit was supported using Claude Code (Opus‐4, Anthropic, San Francisco, CA), with any AI‐generated code being tested before publication.

### 3.2 Data storage and organization

Copick organizes cryoET data hierarchically into experimental runs, where each run groups tomograms and annotations derived from a single region of interest. Within each run, tomographic volumes and feature maps are indexed by voxel spacing, while point annotations and meshes are stored in angstrom coordinates to maintain binning‐independent spatial referencing. Orientations are represented as rotation matrices to avoid ambiguities inherent in Euler angle conventions. Point annotations are stored as JSON files; meshes use binary GL Transmission Format (GLB) format for compatibility with standard 3D tools. Reference density maps for pickable object types are stored separately in the “Objects” directory.

Tomographic data are stored in OME‐Zarr format using 256^3^ voxel chunks with LZ4 compression. Each tomogram includes a three‐level resolution pyramid (full resolution, 2×, and 4× binned). Voxel spacing is encoded as scale transformations in the OME‐Zarr metadata, enabling automatic coordinate conversion between voxel and physical space. Chunk dimensions and compression settings are configurable through the Python API. Image data can be imported from and exported to MRC‐format (Cheng et al., 2015), EM‐format (Nickell et al., 2005) and tagged image file format (TIFF). Tomograms and feature volumes are stored with 32‐bit floating point precision to maximize compatibility with visualization tools. As copick supports both single and multilabel segmentations, semantic segmentations are stored as 8‐bit integer values.

Projects support partitioning into static and mutable layers, configured through the JSON project file. This enables multiple users to share read‐only access to reference data while storing individual annotations separately, preventing conflicts in collaborative workflows. Data models are validated using Pydantic schemas during read and write operations.

### 3.3 Geometry conventions

All spatial positions in copick are expressed in physical coordinates with units of Angstrom, measured from the corner of the tomogram volume using zero‐based indexing in a right‐handed coordinate system. This convention applies uniformly to pick positions, mesh vertices, and the translation components of transformation matrices, ensuring that all annotation types share a common spatial reference frame independent of tomogram binning.

Pick orientations are represented as 4 × 4 homogeneous transformation matrices T that map points from the particle's local coordinate frame to tomogram space: $p_{\text{tomo}}=T\;p_{\mathrm{ref}}$. The rotation submatrix $R\in \mathrm{SO}\left(3\right)$ encodes the particle orientation and the translation component captures any positional refinement shifts relative to the stored pick location after rotation of the reference particle into tomogram frame. Copick implements import and export utilities for the formats used by Relion (Burt et al., 2024), Dynamo, and the TOM toolbox (Nickell et al., 2005), as well as generic comma‐separated value (CSV) import. Import and export utilities include the particle orientations.

Volumetric data, including tomograms and segmentations, are stored as 3D arrays in *ZYX* axis order following the C‐contiguous (row‐major) convention standard in Python and NumPy. The relationship between array indices $\left(i_{z},i_{y},i_{x}\right)$ and physical coordinates is mediated by the isotropic voxel spacing s (in Å/voxel): $\left(x,y,z\right)=\left(i_{x}\times s,i_{y}\times s,i_{z}\times s\right)$. This mapping is encoded as a scale transformation in the OME‐Zarr (Moore et al., 2023) metadata, enabling automatic coordinate conversion between voxel and physical space.

Meshes representing surface annotations are stored in GLB format with vertex coordinates in Angstrom, consistent with the physical coordinate system used for picks and tomograms.

### 3.4 Visualization plugins

The ChimeraX‐copick plugin extends UCSF ChimeraX (Meng et al., 2023) through the ChimeraX‐ArtiaX (Ermel et al., 2022) framework. A standalone ChimeraX‐OME‐Zarr plugin (Ermel, 2025a) was developed to enable reading of multi‐resolution OME‐Zarr data; while developed for copick, this plugin provides general‐purpose OME‐Zarr support for ChimeraX. Pick data are saved automatically when the user switches between particle lists. User interface components shared between visualization plugins, including the tomogram gallery view, are provided by the copick‐shared‐ui library (Ermel, 2025c).

The napari‐copick plugin integrates with napari's native layer system, presenting tomograms as image layers, particle picks as points layers, and segmentations as labels layers. When saving segmentations, the plugin automatically scales data to the specified voxel spacing and generates multi‐resolution OME‐Zarr representations. Both plugins are initialized by providing the path to a copick configuration file, after which all data access is managed through the copick API.

### 3.5 Command‐line toolkit and MCP interface

Two companion packages extend the core copick API with command‐line operations. copick‐utils (Harrington, Schwartz, et al., 2025) provides annotation manipulation including bidirectional format conversion between picks, segmentations, and meshes; Boolean and spatial filtering operations; sampling of points on mesh surfaces; and processing functions such as connected component analysis, skeletonization, and size filtering. copick‐torch (Harrington, Ermel, & Schwartz, 2025) adds PyTorch‐dependent functionality including dataset classes for training patch extraction and a wrapper for running MemBrain‐seg (Lamm et al., 2024) on copick datasets. A complete listing of available commands is provided in Table S1.

The command‐line interface was built using Click with a plugin architecture that allows external packages to register additional commands. The copick‐mcp package (Ermel & Harrington, 2025) exposes these commands to LLMs through a MCP server implemented using fastmcp. The server provides two categories of tools: data exploration tools for read‐only browsing of project contents, and CLI introspection tools that dynamically discover available commands and validate command syntax using Click's native parsing. Registered CLI commands are automatically exposed to the MCP server through introspection, enabling natural language access to new operations without requiring separate implementation.

## 4 DISCUSSION

The development of copick addresses challenges in collaborative cryoET data analysis that have emerged as the field transitions from producing tens of tomograms per study to generating thousands of volumes within days. Modern data acquisition pipelines now routinely generate datasets that exceed the analysis capacity of individual researchers or even research groups (Eisenstein et al., 2023, 2024), creating a need for tools that support scalable, collaborative annotation workflows. Manual annotation of macromolecules within cryoET datasets represents a persistent bottleneck, with ground truth annotation for large‐scale machine learning challenges requiring months of effort from entire research teams (Peck et al., 2025). The copick ecosystem addresses these challenges through three complementary mechanisms: standardized data organization and access, integration with existing visualization tools, and agentic AI‐enabled workflow generation for annotation processing and validation.

### 4.1 Standardization and interoperability

The cryoET field currently faces challenges similar to those encountered by single particle analysis several years ago, where file formats and metadata conventions varied widely across different software packages. This lack of standardization has made it difficult for researchers to combine workflows from different tools or to share datasets across institutions. By implementing a storage‐agnostic API that abstracts underlying filesystem details, copick enables seamless integration of data stored across local systems, HPC environments, cloud platforms, and public repositories. This approach complements ongoing efforts to establish metadata standards for cryoET data, including initiatives by the Electron Microscopy Public Image Archive (EMPIAR) (Iudin et al., 2023) and the CZ cryoET data portal (Ermel et al., 2024) to develop community‐wide formatting and metadata conventions.

The adoption of OME‐Zarr (Moore et al., 2023) as the storage format provides several advantages beyond multi‐resolution visualization. The format's chunked architecture enables partial data loading that is particularly valuable when working with remote data, reducing bandwidth requirements and enabling responsive interaction with datasets too large to transfer entirely. Moreover, OME‐Zarr's adoption across multiple scientific domains—from bioimaging to genomics to earth observation—suggests that tools developed for copick datasets may have broader applicability as the format continues to mature. The current OME‐Zarr specification's evolution toward supporting *n*‐dimensional arrays will further expand the types of cryoET‐derived data that can be efficiently stored and accessed.

While OME‐Zarr adoption is accelerating across the broader bioimaging community, the majority of established cryoET processing tools continue to rely on MRC format for input and output. To maintain compatibility with these existing pipelines, copick includes import and export utilities for MRC (Cheng et al., 2015), EM (Nickell et al., 2005), and TIFF formats, allowing researchers to convert between OME‐Zarr and conventional formats as needed. This approach enables copick to serve as a bridge between OME‐Zarr‐based collaborative workflows and the existing MRC‐centric cryoET software ecosystem without requiring up‐ or downstream tools to adopt new formats.

### 4.2 Natural language interfaces for scientific workflows

The MCP integration enables LLMs to serve as autonomous agents that plan, observe, and iterate on annotation workflows based on natural language specifications. This paradigm shift eliminates the disconnect between biological understanding and technical implementation—researchers express biological constraints directly (“mitochondrial cristae must be contained within the outer membrane”) rather than translating to code or commands themselves. Agents adapt pipelines to edge cases by querying available operations and composing them dynamically.

Agentic execution facilitates knowledge transfer by enabling all team members to specify and modify validation logic through natural language, democratizing computational capabilities beyond traditionally skilled programmers. However, reproducibility requires inspectable, version‐controlled pipelines. Copick addresses this by generating executable bash scripts that researchers can review and archive, with future development targeting formal pipeline specifications and automated validation tools.

### 4.3 Limitations and future directions

Several limitations of the current implementation warrant discussion. The visualization plugins do not yet support editing of volumetric segmentations or mesh annotations within ChimeraX (Meng et al., 2023), requiring researchers to switch to napari (Sofroniew et al., 2025) for segmentation refinement. Future development will focus on providing more complete editing capabilities across both platforms. The command‐line toolkit's operations are currently optimized for point annotations and segmentations; extension to additional data types such as oriented surfaces or parametric curve representations would expand the range of biological structures that can be efficiently analyzed.

The MCP‐based pipeline generation relies on language models' ability to correctly interpret biological constraints and select appropriate operations. While testing has demonstrated effective pipeline generation for common validation scenarios, edge cases and complex multi‐step workflows may require iteration or manual refinement. Continued development of the command‐line toolkit's documentation and examples will improve the language models' ability to generate accurate pipelines. Additionally, the development of validation tools that can verify generated pipelines against expected behaviors would increase confidence in automated pipeline construction.

Looking forward, the copick ecosystem is positioned to support emerging trends in cryoET analysis. The increasing resolution of cryoET data and improvements in sample preparation are enabling visual proteomics approaches that aim to identify and localize many more protein complexes within cells (Kelley et al., 2024; Last et al., 2025). Comprehensive annotation efforts will require coordinated work across multiple institutions and the integration of diverse data types, including correlated light and electron microscopy, mass spectrometry, and genomic data. Copick's storage‐agnostic architecture provides a foundation for these multi‐modal integration efforts.

As machine learning methods for cryoET annotation continue to improve, the role of manual annotation may shift from primary data generation to validation and correction of automated predictions. The copick toolkit's support for both human‐in‐the‐loop refinement and automated quality control positions it to facilitate this transition. The integration with public data repositories and standardized data formats will enable the cryoET community to develop benchmark datasets and track progress in automated annotation methods over time, following successful models from other imaging domains.

## AUTHOR CONTRIBUTIONS

**Utz Heinrich Ermel:** Conceptualization; writing – original draft; writing – review and editing; software; visualization; data curation; formal analysis. **Bridget Carragher:** Writing – original draft; writing – review and editing; supervision; funding acquisition.

## Supporting information


**Figure S1.** The architecture and size of copick datasets. (A) Median size and distribution of compressed multi‐scale OME‐Zarr tomograms (32‐bit float) for three depositions on the cryoET data portal compared to the cumulative sizes of the equivalent MRC‐files (B1—no binning, B2—2× binning, B4—4× binning). (B) Median size and distribution of compressed single‐scale OME‐Zarr segmentations (8‐bit integer) for three depositions on the cryoET data portal compared to sizes of the equivalent MRC‐files.

**Table S1.** copick‐utils and copick‐torch CLI Commands.
**Table S2.** MCP pipeline generation. User prompts and responses resulting in the segmentation curation pipeline in Figure 4b.

## Data Availability

Segmentations created and curated for CZ cryoET data portal Dataset 10155 using the agent‐designed pipeline will be made available on the cryoET data portal under deposition ID CZCDP‐10338. Some of the data used in this work was provided by Peck et al. (CZCDP‐10310) (Peck et al., 2025); Ali et al. (CZCDP‐10336); Yu et al. (CZCDP‐10341), Kelley et al. (CZCDP‐10300) (Kelley et al., 2024) and Kaplan et al. (CZCDP‐10032) (Kaplan et al., 2023b). The data are available through the CryoET Data Portal (Ermel et al., 2024). The core copick API and companion libraries are available under MIT license. Source code is hosted at the copick GitHub organization (https://github.com/copick) with the following repositories: copick (core API) (Ermel et al., 2025), copick‐utils (CLI operations) (Harrington, Schwartz, et al., 2025), copick‐torch (PyTorch functionality) (Harrington, Ermel, & Schwartz, 2025), copick‐mcp (MCP server) (Ermel & Harrington, 2025), chimerax‐copick (ChimeraX plugin) (Ermel & Harrington, 2025), napari‐copick (napari plugin) (Harrington & Ermel, 2025), and copick‐shared‐ui (shared UI components) (Ermel, 2025c). The ChimeraX‐OME‐Zarr plugin (Ermel, 2025a) is available at https://github.com/uermel/chimerax-ome-zarr. All packages are installable via pip. The project maintains documentation at https://copick.github.io/copick/ with API reference, usage examples, and tutorials.
