# Supplementary material for: copick: An open dataset interface and toolkit for collaborative annotation and analysis of cryo‐electron tomography data
Source: Protein Sci. 2026 Apr 17;35(5):e70578. doi: 10.1002/pro.70578 (PMC13090584; doi:10.1002/pro.70578)
Supplement: Supplementary file 1 — Figure S1. The architecture and size of copick datasets. (A) Median size and distribution of compressed multi‐scale OME‐Zarr tomograms (32‐bit float) for three depositions on the cryoET data portal compared to the cumulative sizes of the equivalent MRC‐files (B1—no binning, B2—2× binning, B4—4× binning). (B) Median size and distribution of compressed single‐scale OME‐Zarr segmentations (8‐bit integer) for three depositions on the cryoET data portal compared to sizes of the equivalent MRC‐files. [file PRO-35-e70578-s002.docx]

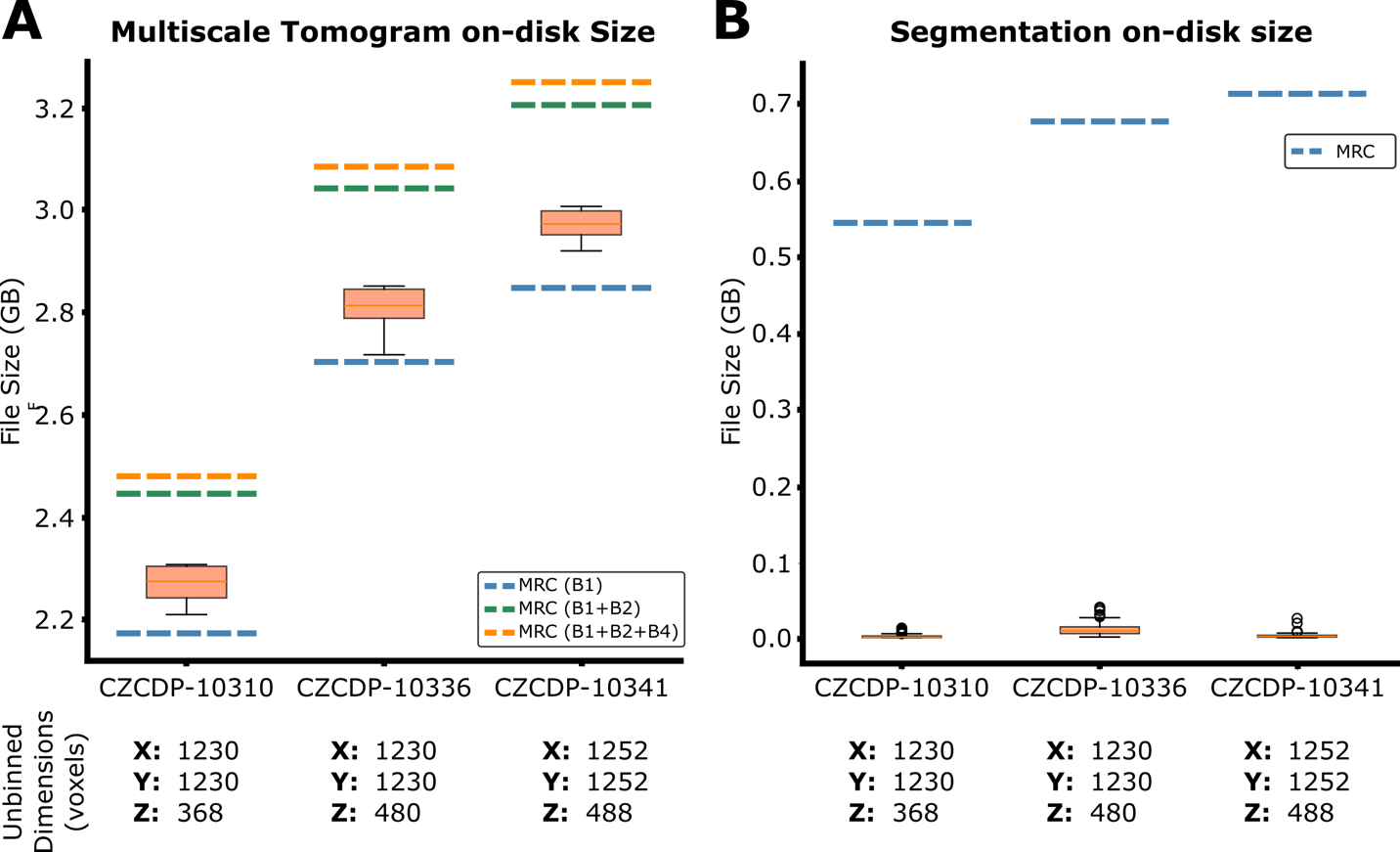


**Figure 1**: **The architecture and size of copick datasets.** **A** Median size and distribution of compressed multi-scale OME-Zarr tomograms (32-bit float) for three depositions on the cryoET data portal compared to the cumulative sizes of the equivalent MRC-files (B1 – no binning, B2 – 2x binning, B4 – 4x binning). **B** Median size and distribution of compressed single-scale OME-Zarr segmentations (8-bit integer) for three depositions on the cryoET data portal compared to sizes of the equivalent MRC-files.
