# Supplementary material for: copick: An open dataset interface and toolkit for collaborative annotation and analysis of cryo‐electron tomography data
Source: Protein Sci. 2026 Apr 17;35(5):e70578. doi: 10.1002/pro.70578 (PMC13090584; doi:10.1002/pro.70578)
Supplement: Supplementary file 2 — Table S1. copick‐utils and copick‐torch CLI Commands. Table S2. MCP pipeline generation. User prompts and responses resulting in the segmentation curation pipeline in Figure 4b. [file PRO-35-e70578-s001.pdf]

Supplementary Table 1: copick-utils and copick-torch CLI Commands.

**Category**	**Command**	**Description**
logical	`clipmesh`	Limit mesh vertices to those within specified distance of a reference surface
logical	`clipseg`	Limit segmentation voxels to those within specified distance of a reference surface
logical	`clippicks`	Limit picks to those within specified distance of a reference surface
logical	`meshop`	Boolean operations between meshes (union, difference, intersection, exclusion, concatenate)
logical	`segop`	Boolean operations between segmentations (union, difference, intersection, exclusion)
logical	`picksin`	Filter picks to include only those inside a reference volume
logical	`picksout`	Filter picks to exclude those inside a reference volume
logical	`enclosed`	Remove segmented regions fully enclosed by another segmentation
convert	`picks2seg`	Convert picks to segmentation volumes by painting spheres at pick coordinates
convert	`seg2picks`	Convert segmentation volumes to picks by extracting component centroids
convert	`seg2mesh`	Convert segmentation volumes to meshes using marching cubes
convert	`mesh2seg`	Convert meshes to segmentation volumes (watertight fill or boundary voxelization)
convert	`picks2mesh`	Convert picks to meshes using convex hull or alpha shapes
convert	`mesh2picks`	Sample picks from meshes (surface, inside, or outside sampling)

**Category**	**Command**	**Description**
convert	`picks2sphere`	Convert picks to sphere meshes
convert	`picks2ellipsoid`	Convert picks to ellipsoid meshes
convert	`picks2plane`	Convert picks to plane meshes
convert	`picks2surface`	Convert picks to 2D surface meshes
process	`separate-components`	Separate connected components into individual segmentations
process	`filter-components`	Filter connected components by size (cubic angstroms)
process	`skeletonize`	3D skeletonization of segmentations
process	`fit-spline`	Fit 3D splines to skeletons and generate oriented picks
process	`hull`	Compute convex hull of meshes
process	`validbox`	Generate valid area box meshes for tomographic reconstructions
process	`downsample`*	Downsample tomographic data
project	`config`	Create and manage copick configuration files
project	`new`	Create copick entities
project	`cp`	Copy/duplicate copick objects by URI
project	`mv`	Move/rename copick objects by URI
project	`rm`	Remove copick objects by URI
project	`sync`	Synchronize data between copick projects
project	`deposit`	Create depositable view of a project using symlinks
project	`stats`	Gather statistics about a copick project

Category	Command	Description
Inference	`membrain-seg`*	Run MemBrain-seg membrane segmentation on copick datasets

*Commands from copick-torch; all others from copick-utils.

Supplementary Table 2: MCP pipeline generation. User prompts and responses resulting in the segmentation curation pipeline in Fig. 4B.

User	I have a copick project that has multiple membrane segmentations from data-portal, a segmentation for cytosole, cell, inclusion-body and flagellum (all from tool napari, session manual-scaled).<br><br>I want you to create a copick pipeline that<br><br>1. combines all available membrane segmentations from the data portal<br>2. limits the membrane segmentation to 200 A from the cell surface<br>3. generates an intermembrane-space segmentation by subtracting cytosole from cell<br>4. excludes pieces of membrane fully surrounded by cytosole from the membrane segmentation<br>5. subtracts the inclusion-body segmentation from the cytosole segmentation<br>6. Ensures that the membrane segmentation does not overlap with cytosole, intermembrane-space or flagellum<br><br>For each final output, can you please set the session-id final-training. Please create a bash script that contains all final commands. Please have the pipeline remove intermediate results.

Claude Opus-4.1	(code below)

Shell

```
#!/bin/bash

# Copick Segmentation Processing Pipeline
# This script processes membrane, cytosole, cell,
inclusion-body, and flagellum segmentations

# Configuration
CONFIG_PATH="./copick_config.json"  # Update with your actual
config path
VOXEL_SPACING=10.0  # Adjust based on your data

# User/session IDs for input segmentations
INPUT_USER="napari"
INPUT_SESSION="manual-scaled"

# Temporary intermediate user/session IDs
TEMP_USER="temp"

# Final output session ID
FINAL_SESSION="final-training"

# Run names (optional - leave empty to process all runs)
# Add specific run names like: -r "run1" -r "run2"
RUN_OPTS=""

echo "=========================================="
echo "Starting Copick Segmentation Pipeline"
echo "=========================================="

# Step 1: Combine all membrane segmentations from data-portal
echo "Step 1: Combining all membrane segmentations from
data-portal..."
copick logical segop \
    -c "$CONFIG_PATH" \
    --operation union \
    -vs $VOXEL_SPACING \
    -i "membrane:data-portal/*@$VOXEL_SPACING" \
    -o "membrane:$TEMP_USER/combined-membranes@$VOXEL_SPACING" \
    $RUN_OPTS
```

```
# Step 2: Limit membrane segmentation to 200 Angstroms from cell
surface
echo "Step 2: Limiting membrane to 200 Angstroms from cell
surface..."
copick logical clipseg \
    -c "$CONFIG_PATH" \
    -i "membrane:$TEMP_USER/combined-membranes@$VOXEL_SPACING" \
    -rs "cell:$INPUT_USER/$INPUT_SESSION@$VOXEL_SPACING" \
    -o "membrane:$TEMP_USER/membrane-clipped@$VOXEL_SPACING" \
    --max-distance 200.0 \
    $RUN_OPTS

# Step 3: Generate intermembrane-space segmentation (cell -
cytosole)
echo "Step 3: Generating intermembrane-space segmentation..."
copick logical segop \
    -c "$CONFIG_PATH" \
    --operation difference \
    -vs $VOXEL_SPACING \
    -i "cell:$INPUT_USER/$INPUT_SESSION@$VOXEL_SPACING" \
    -i "cytosole:$INPUT_USER/$INPUT_SESSION@$VOXEL_SPACING" \
    -o
"intermembrane-space:$TEMP_USER/intermembrane@$VOXEL_SPACING" \
    $RUN_OPTS

# Step 4: Remove membrane pieces fully surrounded by cytosole
echo "Step 4: Removing membrane pieces fully surrounded by
cytosole..."
copick logical enclosed \
    -c "$CONFIG_PATH" \
    -vs $VOXEL_SPACING \
    -i1 "membrane:$TEMP_USER/membrane-clipped@$VOXEL_SPACING" \
    -i2 "cytosole:$INPUT_USER/$INPUT_SESSION@$VOXEL_SPACING" \
    -o "membrane:$TEMP_USER/membrane-cleaned@$VOXEL_SPACING" \
    --margin 2 \
    $RUN_OPTS

# Step 5: Subtract inclusion-body from cytosole
echo "Step 5: Subtracting inclusion-body from cytosole..."
copick logical segop \
```

```
    -c "$CONFIG_PATH" \
    --operation difference \
    -vs $VOXEL_SPACING \
    -i "cytosole:$INPUT_USER/$INPUT_SESSION@$VOXEL_SPACING" \
    -i
"inclusion-body:$INPUT_USER/$INPUT_SESSION@$VOXEL_SPACING" \
    -o
"cytosole:$TEMP_USER/cytosole-no-inclusion@$VOXEL_SPACING" \
    $RUN_OPTS

# Step 6a: Remove cytosole overlap from membrane
echo "Step 6a: Removing cytosole overlap from membrane..."
copick logical segop \
    -c "$CONFIG_PATH" \
    --operation difference \
    -vs $VOXEL_SPACING \
    -i "membrane:$TEMP_USER/membrane-cleaned@$VOXEL_SPACING" \
    -i
"cytosole:$TEMP_USER/cytosole-no-inclusion@$VOXEL_SPACING" \
    -o "membrane:$TEMP_USER/membrane-no-cytosole@$VOXEL_SPACING"
\
    $RUN_OPTS

# Step 6b: Remove intermembrane-space overlap from membrane
echo "Step 6b: Removing intermembrane-space overlap from
membrane..."
copick logical segop \
    -c "$CONFIG_PATH" \
    --operation difference \
    -vs $VOXEL_SPACING \
    -i "membrane:$TEMP_USER/membrane-no-cytosole@$VOXEL_SPACING"
\
    -i
"intermembrane-space:$TEMP_USER/intermembrane@$VOXEL_SPACING" \
    -o "membrane:$TEMP_USER/membrane-no-ims@$VOXEL_SPACING" \
    $RUN_OPTS

# Step 6c: Remove flagellum overlap from membrane
echo "Step 6c: Removing flagellum overlap from membrane..."
copick logical segop \
```

```
    -c "$CONFIG_PATH" \
    --operation difference \
    -vs $VOXEL_SPACING \
    -i "membrane:$TEMP_USER/membrane-no-ims@$VOXEL_SPACING" \
    -i "flagellum:$INPUT_USER/$INPUT_SESSION@$VOXEL_SPACING" \
    -o "membrane:$TEMP_USER/membrane-final-temp@$VOXEL_SPACING"
\
    $RUN_OPTS

# ========================================
# Move final results to final-training session
# ========================================

echo ""
echo "=========================================="
echo "Moving results to final-training session..."
echo "=========================================="

# Move final membrane segmentation
copick mv segmentation \
    -c "$CONFIG_PATH" \
    "membrane:$TEMP_USER/membrane-final-temp@$VOXEL_SPACING" \
    "membrane:$INPUT_USER/$FINAL_SESSION@$VOXEL_SPACING" \
    $RUN_OPTS

# Move final cytosole segmentation (without inclusion-body)
copick mv segmentation \
    -c "$CONFIG_PATH" \
    "cytosole:$TEMP_USER/cytosole-no-inclusion@$VOXEL_SPACING" \
    "cytosole:$INPUT_USER/$FINAL_SESSION@$VOXEL_SPACING" \
    $RUN_OPTS

# Move final intermembrane-space segmentation
copick mv segmentation \
    -c "$CONFIG_PATH" \

"intermembrane-space:$TEMP_USER/intermembrane@$VOXEL_SPACING" \

"intermembrane-space:$INPUT_USER/$FINAL_SESSION@$VOXEL_SPACING"
\
```

	(code below)

```
    $RUN_OPTS

# Copy original segmentations that don't need modification to
final-training
echo "Copying unchanged segmentations to final-training..."

# Copy cell segmentation
copick cp segmentation \
    -c "$CONFIG_PATH" \
    "cell:$INPUT_USER/$INPUT_SESSION@$VOXEL_SPACING" \
    "cell:$INPUT_USER/$FINAL_SESSION@$VOXEL_SPACING" \
    $RUN_OPTS

# Copy flagellum segmentation
copick cp segmentation \
    -c "$CONFIG_PATH" \
    "flagellum:$INPUT_USER/$INPUT_SESSION@$VOXEL_SPACING" \
    "flagellum:$INPUT_USER/$FINAL_SESSION@$VOXEL_SPACING" \
    $RUN_OPTS

# Copy inclusion-body segmentation
copick cp segmentation \
    -c "$CONFIG_PATH" \
    "inclusion-body:$INPUT_USER/$INPUT_SESSION@$VOXEL_SPACING" \
    "inclusion-body:$INPUT_USER/$FINAL_SESSION@$VOXEL_SPACING" \
    $RUN_OPTS

# ========================================
# Clean up intermediate results
# ========================================

echo ""
echo "==========================================="
echo "Cleaning up intermediate results..."
echo "==========================================="

# Remove all temporary intermediate segmentations
# Note: Using pattern matching to remove all temp user
segmentations
```

	```<br>copick rm segmentation \<br>-c "$CONFIG_PATH" \<br>--force \<br>"membrane:$TEMP_USER/*@$VOXEL_SPACING" \<br>$RUN_OPTS<br><br>copick rm segmentation \<br>-c "$CONFIG_PATH" \<br>--force \<br>"cytosole:$TEMP_USER/*@$VOXEL_SPACING" \<br>$RUN_OPTS<br><br>copick rm segmentation \<br>-c "$CONFIG_PATH" \<br>--force \<br>"intermembrane-space:$TEMP_USER/*@$VOXEL_SPACING" \<br>$RUN_OPTS<br><br>echo ""<br>echo "=="<br>echo "Pipeline Complete!"<br>echo "=="<br>echo ""<br>echo "Final outputs with session ID '$FINAL_SESSION':"<br>echo " - membrane (limited to 200Å from cell, no overlaps)"<br>echo " - cytosole (inclusion-body removed)"<br>echo " - intermembrane-space (cell - cytosole)"<br>echo " - cell (original)"<br>echo " - flagellum (original)"<br>echo " - inclusion-body (original)"<br>echo ""<br>echo "All intermediate results have been removed."<br>```
User	for step 6 a, b, c I think you misunderstood: I want to remove the overlap with membrane from cytosole, intermembrane-space and flagellum, not remove it from membrane. please update the pipeline
Claude	

Opus-4.1*

Shell

```
#!/bin/bash

# Copick Segmentation Processing Pipeline
# This script processes membrane, cytosole, cell, inclusion-body, and
flagellum segmentations

# Configuration
CONFIG_PATH="./copick_config.json"  # Update with your actual config path
VOXEL_SPACING=10.0  # Adjust based on your data

# User/session IDs for input segmentations
INPUT_USER="napari"
INPUT_SESSION="manual-scaled"

# Temporary intermediate user/session IDs
TEMP_USER="temp"

# Final output session ID
FINAL_SESSION="final-training"

# Run names (optional - leave empty to process all runs)
# Add specific run names like: -r "run1" -r "run2"
RUN_OPTS=""

echo "=========================================="
echo "Starting Copick Segmentation Pipeline"
echo "=========================================="

# Step 1: Combine all membrane segmentations from data-portal
echo "Step 1: Combining all membrane segmentations from data-portal..."
copick logical segop \
    -c "$CONFIG_PATH" \
    --operation union \
    -vs $VOXEL_SPACING \
    -i "membrane:data-portal/*@$VOXEL_SPACING" \
    -o "membrane:$TEMP_USER/combined-membranes@$VOXEL_SPACING" \
    $RUN_OPTS

# Step 2: Limit membrane segmentation to 200 Angstroms from cell surface
echo "Step 2: Limiting membrane to 200 Angstroms from cell surface..."
copick logical clipseg \
    -c "$CONFIG_PATH" \
    -i "membrane:$TEMP_USER/combined-membranes@$VOXEL_SPACING" \
    -rs "cell:$INPUT_USER/$INPUT_SESSION@$VOXEL_SPACING" \
    -o "membrane:$TEMP_USER/membrane-clipped@$VOXEL_SPACING" \
    --max-distance 200.0 \
    $RUN_OPTS

# Step 3: Generate intermembrane-space segmentation (cell - cytosole)
echo "Step 3: Generating intermembrane-space segmentation..."
```

	```<br>copick logical segop \<br>-c "$CONFIG_PATH" \<br>--operation difference \<br>-vs $VOXEL_SPACING \<br>-i "cell:$INPUT_USER/$INPUT_SESSION@$VOXEL_SPACING" \<br>-i "cytosole:$INPUT_USER/$INPUT_SESSION@$VOXEL_SPACING" \<br>-o "intermembrane-space:$TEMP_USER/intermembrane@$VOXEL_SPACING" \<br>$RUN_OPTS<br>```

```
copick logical segop \
    -c "$CONFIG_PATH" \
    --operation difference \
    -vs $VOXEL_SPACING \
    -i "cell:$INPUT_USER/$INPUT_SESSION@$VOXEL_SPACING" \
    -i "cytosole:$INPUT_USER/$INPUT_SESSION@$VOXEL_SPACING" \
    -o "intermembrane-space:$TEMP_USER/intermembrane@$VOXEL_SPACING" \
    $RUN_OPTS

# Step 4: Remove membrane pieces fully surrounded by cytosole
echo "Step 4: Removing membrane pieces fully surrounded by cytosole..."
copick logical enclosed \
    -c "$CONFIG_PATH" \
    -vs $VOXEL_SPACING \
    -i1 "membrane:$TEMP_USER/membrane-clipped@$VOXEL_SPACING" \
    -i2 "cytosole:$INPUT_USER/$INPUT_SESSION@$VOXEL_SPACING" \
    -o "membrane:$TEMP_USER/membrane-cleaned@$VOXEL_SPACING" \
    --margin 2 \
    $RUN_OPTS

# Step 5: Subtract inclusion-body from cytosole
echo "Step 5: Subtracting inclusion-body from cytosole..."
copick logical segop \
    -c "$CONFIG_PATH" \
    --operation difference \
    -vs $VOXEL_SPACING \
    -i "cytosole:$INPUT_USER/$INPUT_SESSION@$VOXEL_SPACING" \
    -i "inclusion-body:$INPUT_USER/$INPUT_SESSION@$VOXEL_SPACING" \
    -o "cytosole:$TEMP_USER/cytosole-no-inclusion@$VOXEL_SPACING" \
    $RUN_OPTS

# Step 6a: Remove membrane overlap from cytosole
echo "Step 6a: Removing membrane overlap from cytosole..."
copick logical segop \
    -c "$CONFIG_PATH" \
    --operation difference \
    -vs $VOXEL_SPACING \
    -i "cytosole:$TEMP_USER/cytosole-no-inclusion@$VOXEL_SPACING" \
    -i "membrane:$TEMP_USER/membrane-cleaned@$VOXEL_SPACING" \
    -o "cytosole:$TEMP_USER/cytosole-no-membrane@$VOXEL_SPACING" \
    $RUN_OPTS

# Step 6b: Remove membrane overlap from intermembrane-space
echo "Step 6b: Removing membrane overlap from intermembrane-space..."
copick logical segop \
    -c "$CONFIG_PATH" \
    --operation difference \
    -vs $VOXEL_SPACING \
    -i "intermembrane-space:$TEMP_USER/intermembrane@$VOXEL_SPACING" \
    -i "membrane:$TEMP_USER/membrane-cleaned@$VOXEL_SPACING" \
```

```
    -o
"intermembrane-space:$TEMP_USER/intermembrane-no-membrane@$VOXEL_SPACING" \
    $RUN_OPTS

# Step 6c: Remove membrane overlap from flagellum
echo "Step 6c: Removing membrane overlap from flagellum..."
copick logical segop \
    -c "$CONFIG_PATH" \
    --operation difference \
    -vs $VOXEL_SPACING \
    -i "flagellum:$INPUT_USER/$INPUT_SESSION@$VOXEL_SPACING" \
    -i "membrane:$TEMP_USER/membrane-cleaned@$VOXEL_SPACING" \
    -o "flagellum:$TEMP_USER/flagellum-no-membrane@$VOXEL_SPACING" \
    $RUN_OPTS

# ========================================
# Move final results to final-training session
# ========================================

echo ""
echo "=========================================="
echo "Moving results to final-training session..."
echo "=========================================="

# Move final membrane segmentation
copick mv segmentation \
    -c "$CONFIG_PATH" \
    "membrane:$TEMP_USER/membrane-cleaned@$VOXEL_SPACING" \
    "membrane:$INPUT_USER/$FINAL_SESSION@$VOXEL_SPACING" \
    $RUN_OPTS

# Move final cytosole segmentation (without inclusion-body and membrane
overlap)
copick mv segmentation \
    -c "$CONFIG_PATH" \
    "cytosole:$TEMP_USER/cytosole-no-membrane@$VOXEL_SPACING" \
    "cytosole:$INPUT_USER/$FINAL_SESSION@$VOXEL_SPACING" \
    $RUN_OPTS

# Move final intermembrane-space segmentation (without membrane overlap)
copick mv segmentation \
    -c "$CONFIG_PATH" \
    "intermembrane-space:$TEMP_USER/intermembrane-no-membrane@$VOXEL_SPACING"
\
    "intermembrane-space:$INPUT_USER/$FINAL_SESSION@$VOXEL_SPACING" \
    $RUN_OPTS

# Move final flagellum segmentation (without membrane overlap)
copick mv segmentation \
    -c "$CONFIG_PATH" \
```

```
    "flagellum:$TEMP_USER/flagellum-no-membrane@$VOXEL_SPACING" \
    "flagellum:$INPUT_USER/$FINAL_SESSION@$VOXEL_SPACING" \
    $RUN_OPTS

# Copy original segmentations that don't need modification to final-training
echo "Copying unchanged segmentations to final-training..."

# Copy cell segmentation
copick cp segmentation \
    -c "$CONFIG_PATH" \
    "cell:$INPUT_USER/$INPUT_SESSION@$VOXEL_SPACING" \
    "cell:$INPUT_USER/$FINAL_SESSION@$VOXEL_SPACING" \
    $RUN_OPTS

# Copy inclusion-body segmentation
copick cp segmentation \
    -c "$CONFIG_PATH" \
    "inclusion-body:$INPUT_USER/$INPUT_SESSION@$VOXEL_SPACING" \
    "inclusion-body:$INPUT_USER/$FINAL_SESSION@$VOXEL_SPACING" \
    $RUN_OPTS

# ========================================
# Clean up intermediate results
# ========================================

echo ""
echo "=========================================="
echo "Cleaning up intermediate results..."
echo "=========================================="

# Remove all temporary intermediate segmentations
# Note: Using pattern matching to remove all temp user segmentations

copick rm segmentation \
    -c "$CONFIG_PATH" \
    --force \
    "membrane:$TEMP_USER/*@$VOXEL_SPACING" \
    $RUN_OPTS

copick rm segmentation \
    -c "$CONFIG_PATH" \
    --force \
    "cytosole:$TEMP_USER/*@$VOXEL_SPACING" \
    $RUN_OPTS

copick rm segmentation \
    -c "$CONFIG_PATH" \
    --force \
    "intermembrane-space:$TEMP_USER/*@$VOXEL_SPACING" \
    $RUN_OPTS
```

<table>
<tr><td></td><td>

```
copick rm segmentation \
    -c "$CONFIG_PATH" \
    --force \
    "flagellum:$TEMP_USER/*@$VOXEL_SPACING" \
    $RUN_OPTS

echo ""
echo "=========================================="
echo "Pipeline Complete!"
echo "=========================================="
echo ""
echo "Final outputs with session ID '$FINAL_SESSION':"
echo "  - membrane (limited to 200Å from cell, enclosed pieces removed)"
echo "  - cytosole (inclusion-body removed, no membrane overlap)"
echo "  - intermembrane-space (cell - cytosole, no membrane overlap)"
echo "  - flagellum (no membrane overlap)"
echo "  - cell (original)"
echo "  - inclusion-body (original)"
echo ""
echo "All intermediate results have been removed."
```

</td></tr>
<tr><td>User</td><td>I ran into another issue: for some runs, there is no inclusion-body segmentation. this causes the cytosole-no-inclusion segmentation to be absent and thus not being copied. can you find a solution where this is not the case? (e.g. an additional copy before the subtraction?)</td></tr>
<tr><td>Claude Opus-4.1*</td><td>

Shell

```
#!/bin/bash

# Copick Segmentation Processing Pipeline
# This script processes membrane, cytosole, cell, inclusion-body, and
flagellum segmentations

# Configuration
CONFIG_PATH="./copick_config.json"  # Update with your actual config path
VOXEL_SPACING=10.0  # Adjust based on your data

# User/session IDs for input segmentations
INPUT_USER="napari"
INPUT_SESSION="manual-scaled"

# Temporary intermediate user/session IDs
TEMP_USER="temp"
```

</td></tr>
</table>

```
# Final output session ID
FINAL_SESSION="final-training"

# Run names (optional - leave empty to process all runs)
# Add specific run names like: -r "run1" -r "run2"
RUN_OPTS=""

echo "=========================================="
echo "Starting Copick Segmentation Pipeline"
echo "=========================================="

# Step 1: Combine all membrane segmentations from data-portal
echo "Step 1: Combining all membrane segmentations from data-portal..."
copick logical segop \
    -c "$CONFIG_PATH" \
    --operation union \
    -vs $VOXEL_SPACING \
    -i "membrane:data-portal/*@$VOXEL_SPACING" \
    -o "membrane:$TEMP_USER/combined-membranes@$VOXEL_SPACING" \
    $RUN_OPTS

# Step 2: Limit membrane segmentation to 200 Angstroms from cell surface
echo "Step 2: Limiting membrane to 200 Angstroms from cell surface..."
copick logical clipseg \
    -c "$CONFIG_PATH" \
    -i "membrane:$TEMP_USER/combined-membranes@$VOXEL_SPACING" \
    -rs "cell:$INPUT_USER/$INPUT_SESSION@$VOXEL_SPACING" \
    -o "membrane:$TEMP_USER/membrane-clipped@$VOXEL_SPACING" \
    --max-distance 200.0 \
    $RUN_OPTS

# Step 3: Generate intermembrane-space segmentation (cell - cytosole)
echo "Step 3: Generating intermembrane-space segmentation..."
copick logical segop \
    -c "$CONFIG_PATH" \
    --operation difference \
    -vs $VOXEL_SPACING \
    -i "cell:$INPUT_USER/$INPUT_SESSION@$VOXEL_SPACING" \
    -i "cytosole:$INPUT_USER/$INPUT_SESSION@$VOXEL_SPACING" \
    -o "intermembrane-space:$TEMP_USER/intermembrane@$VOXEL_SPACING" \
    $RUN_OPTS

# Step 4: Remove membrane pieces fully surrounded by cytosole
echo "Step 4: Removing membrane pieces fully surrounded by cytosole..."
copick logical enclosed \
    -c "$CONFIG_PATH" \
    -vs $VOXEL_SPACING \
    -i1 "membrane:$TEMP_USER/membrane-clipped@$VOXEL_SPACING" \
    -i2 "cytosole:$INPUT_USER/$INPUT_SESSION@$VOXEL_SPACING" \
    -o "membrane:$TEMP_USER/membrane-cleaned@$VOXEL_SPACING" \
```

```
    --margin 2 \
    $RUN_OPTS

# Step 5: Subtract inclusion-body from cytosole (if it exists)
echo "Step 5: Processing cytosole (removing inclusion-body if present)..."

# First, copy cytosole to the temporary location
echo "  Copying cytosole to temporary location..."
copick cp segmentation \
    -c "$CONFIG_PATH" \
    "cytosole:$INPUT_USER/$INPUT_SESSION@$VOXEL_SPACING" \
    "cytosole:$TEMP_USER/cytosole-no-inclusion@$VOXEL_SPACING" \
    $RUN_OPTS

# Try to subtract inclusion-body if it exists (this will overwrite the copy if
successful)
echo "  Attempting to subtract inclusion-body from cytosole..."
copick logical segop \
    -c "$CONFIG_PATH" \
    --operation difference \
    -vs $VOXEL_SPACING \
    -i "cytosole:$INPUT_USER/$INPUT_SESSION@$VOXEL_SPACING" \
    -i "inclusion-body:$INPUT_USER/$INPUT_SESSION@$VOXEL_SPACING" \
    -o "cytosole:$TEMP_USER/cytosole-no-inclusion@$VOXEL_SPACING" \
    --overwrite \
    $RUN_OPTS 2>/dev/null || echo "  Note: No inclusion-body found for
some/all runs - using original cytosole"

# Step 6a: Remove membrane overlap from cytosole
echo "Step 6a: Removing membrane overlap from cytosole..."
copick logical segop \
    -c "$CONFIG_PATH" \
    --operation difference \
    -vs $VOXEL_SPACING \
    -i "cytosole:$TEMP_USER/cytosole-no-inclusion@$VOXEL_SPACING" \
    -i "membrane:$TEMP_USER/membrane-cleaned@$VOXEL_SPACING" \
    -o "cytosole:$TEMP_USER/cytosole-no-membrane@$VOXEL_SPACING" \
    $RUN_OPTS

# Step 6b: Remove membrane overlap from intermembrane-space
echo "Step 6b: Removing membrane overlap from intermembrane-space..."
copick logical segop \
    -c "$CONFIG_PATH" \
    --operation difference \
    -vs $VOXEL_SPACING \
    -i "intermembrane-space:$TEMP_USER/intermembrane@$VOXEL_SPACING" \
    -i "membrane:$TEMP_USER/membrane-cleaned@$VOXEL_SPACING" \
    -o
"intermembrane-space:$TEMP_USER/intermembrane-no-membrane@$VOXEL_SPACING" \
    $RUN_OPTS
```

```
# Step 6c: Remove membrane overlap from flagellum
echo "Step 6c: Removing membrane overlap from flagellum..."
copick logical segop \
    -c "$CONFIG_PATH" \
    --operation difference \
    -vs $VOXEL_SPACING \
    -i "flagellum:$INPUT_USER/$INPUT_SESSION@$VOXEL_SPACING" \
    -i "membrane:$TEMP_USER/membrane-cleaned@$VOXEL_SPACING" \
    -o "flagellum:$TEMP_USER/flagellum-no-membrane@$VOXEL_SPACING" \
    $RUN_OPTS

# ========================================
# Move final results to final-training session
# ========================================

echo ""
echo "=========================================="
echo "Moving results to final-training session..."
echo "=========================================="

# Move final membrane segmentation
copick mv segmentation \
    -c "$CONFIG_PATH" \
    "membrane:$TEMP_USER/membrane-cleaned@$VOXEL_SPACING" \
    "membrane:$INPUT_USER/$FINAL_SESSION@$VOXEL_SPACING" \
    $RUN_OPTS

# Move final cytosole segmentation (without inclusion-body and membrane
overlap)
copick mv segmentation \
    -c "$CONFIG_PATH" \
    "cytosole:$TEMP_USER/cytosole-no-membrane@$VOXEL_SPACING" \
    "cytosole:$INPUT_USER/$FINAL_SESSION@$VOXEL_SPACING" \
    $RUN_OPTS

# Move final intermembrane-space segmentation (without membrane overlap)
copick mv segmentation \
    -c "$CONFIG_PATH" \
    "intermembrane-space:$TEMP_USER/intermembrane-no-membrane@$VOXEL_SPACING"
\
    "intermembrane-space:$INPUT_USER/$FINAL_SESSION@$VOXEL_SPACING" \
    $RUN_OPTS

# Move final flagellum segmentation (without membrane overlap)
copick mv segmentation \
    -c "$CONFIG_PATH" \
    "flagellum:$TEMP_USER/flagellum-no-membrane@$VOXEL_SPACING" \
    "flagellum:$INPUT_USER/$FINAL_SESSION@$VOXEL_SPACING" \
    $RUN_OPTS
```

	```<br>```

```
# Copy original segmentations that don't need modification to final-training
echo "Copying unchanged segmentations to final-training..."

# Copy cell segmentation
copick cp segmentation \
    -c "$CONFIG_PATH" \
    "cell:$INPUT_USER/$INPUT_SESSION@$VOXEL_SPACING" \
    "cell:$INPUT_USER/$FINAL_SESSION@$VOXEL_SPACING" \
    $RUN_OPTS

# Copy inclusion-body segmentation (if it exists)
echo "Attempting to copy inclusion-body segmentation..."
copick cp segmentation \
    -c "$CONFIG_PATH" \
    "inclusion-body:$INPUT_USER/$INPUT_SESSION@$VOXEL_SPACING" \
    "inclusion-body:$INPUT_USER/$FINAL_SESSION@$VOXEL_SPACING" \
    $RUN_OPTS 2>/dev/null || echo "  Note: No inclusion-body segmentation
found for some/all runs"

# ========================================
# Clean up intermediate results
# ========================================

echo ""
echo "=========================================="
echo "Cleaning up intermediate results..."
echo "=========================================="

# Remove all temporary intermediate segmentations
# Note: Using pattern matching to remove all temp user segmentations

copick rm segmentation \
    -c "$CONFIG_PATH" \
    --force \
    "membrane:$TEMP_USER/*@$VOXEL_SPACING" \
    $RUN_OPTS

copick rm segmentation \
    -c "$CONFIG_PATH" \
    --force \
    "cytosole:$TEMP_USER/*@$VOXEL_SPACING" \
    $RUN_OPTS

copick rm segmentation \
    -c "$CONFIG_PATH" \
    --force \
    "intermembrane-space:$TEMP_USER/*@$VOXEL_SPACING" \
    $RUN_OPTS
```

	```<br>copick rm segmentation \<br>-c "$CONFIG_PATH" \<br>--force \<br>"flagellum:$TEMP_USER/*@$VOXEL_SPACING" \<br>$RUN_OPTS<br><br>echo ""<br>echo "=="<br>echo "Pipeline Complete!"<br>echo "=="<br>echo ""<br>echo "Final outputs with session ID '$FINAL_SESSION':"<br>echo " - membrane (limited to 200Å from cell, enclosed pieces removed)"<br>echo " - cytosole (inclusion-body removed, no membrane overlap)"<br>echo " - intermembrane-space (cell - cytosole, no membrane overlap)"<br>echo " - flagellum (no membrane overlap)"<br>echo " - cell (original)"<br>echo " - inclusion-body (original)"<br>echo ""<br>echo "All intermediate results have been removed."<br>```

* thought process omitted
